# A Multidimensional Reputation Barometer for Public Agencies: A Validated Instrument

**DOI:** 10.1111/puar.13158

**Published:** 2020-02-18

**Authors:** Sjors Overman, Madalina Busuioc, Matthew Wood

**Affiliations:** ^1^ Utrecht University; ^2^ Leiden University; ^3^ University of Sheffield

## Abstract

*Reputation is of growing interest for the study of public bureaucracies*, *but a measurement that can discern between the subdimensions of reputation and is validated on real‐life audiences has remained elusive*. *The authors deductively build*, *test*, *and cross‐validate a survey instrument through two surveys of 2*,*100 key stakeholders of the European Chemicals Agency*, *the European Union chemicals regulator*. *This empirical tool measures an agency's reputation and its building blocks*. *This scale represents an important contribution to reputation literature, as it allows scholars to distinguish and measure which aspects of reputation public organizations are “known for” and build their claim to authority on*, *as well as how the profiles of public organizations differ*. *The authors find that direct stakeholder contact with the agency is necessary for stakeholders to be able to evaluate the separate dimensions of reputation independently*.

Evidence for Practice

- This study equips practitioners with a reputation barometer tailored to the public sector. It allows them to measure the reputation of their organization, in a differentiated fashion, among different stakeholder groups.
- While public organizations increasingly engage in reputation management activities, a potential caveat that emerges from our exercise is that managers might be steering in non‐astute directions. While our study shows that, as for private actors, “performance matters,” procedural and moral aspects also weigh heavily in the eyes of stakeholders when it comes to public regulators.
- To secure a positive organizational image and the authority crucial for public agencies to operate, the performance management turn in the public sector may need to be supplemented by an enhanced organizational attention to procedural and moral aspects.

Informed by Daniel Carpenter's seminal work (2001, 2010), organizational reputation has become a dominant perspective in the study of the bureaucracy over the past decade. The successful cultivation of a strong reputation is a crucial ingredient of regulatory “power,” beyond formal fiat, and a fundamental force for state building, key to our understanding of the role of public administration (Carpenter and Krause 2012). Carpenter (2010, 34) contends that reputation “shape[s] the power of government organizations, and more broadly, the powers of the state.” The value of a reputational approach to the public sector has been ascertained with respect to a broad array of regulatory behaviors, including autonomy building (e.g., Carpenter 2001; Groenleer 2011), practices of turf protection, and bureaucratic cooperation (Maor 2010; Moynihan 2012; Busuioc 2016). These empirical studies confirm the relevance of reputation as an important determinant of bureaucratic behavior.

Yet a persistent, nagging critique lingers: the difficulty of measuring the central construct. The complexity of capturing organizational reputation is perhaps best illustrated by Carpenter's *Reputation and Power* (2010), which draws on a variety of methods—both historical narrative and statistical analysis—and employs varied sources of data including extensive use of archival documents, interviews, and additional sources such as scientific magazines and medical journals. This approach invariably raises the question of how to translate these findings into different contexts and the possibility of testing and replicating—and fundamentally also, of falsifying—the approach and its central predictions in different organizational and administrative contexts.

Reputation measurements exist for the private sector (corporate reputation scales, validated in different organizational and geographic locations), but they are too narrowly focused for use in a public sector environment, an essentially different context that requires a tailored approach: “The reputation management recipe encounters a different context in the public sector than for what it originally was intended” (Wæraas and Byrkjeflot 2012, p. 189). Reputation in the private sector signals a competitive advantage (Hall 1993), linked primarily to the bottom line (profit), whereas in the public sector, other aspects need to be included and gain prominence. The moral dimension of reputation, for example, is an indispensable aspect of reputation in the public context (Carpenter 2001). Within this context, reputation is a crucial ingredient, among others, of bureaucratic autonomy and authority (Carpenter 2001, 2010; Carpenter and Krause 2015), and it has fundamental implications for political control (Bertelli and Busuioc 2018).

Public organizations compete, too, like their private counterparts (e.g., over resources, clients, and qualified employees; see Bankins and Waterhouse 2018; Wæraas and Byrkjeflot 2012), and they face challenges of demonstrating uniqueness and added value over and above other organizations to secure stakeholder and clientele support, employee loyalty, and public support more broadly (Carpenter 2001; Groenleer 2011; Wæraas and Byrkjeflot 2012; Wilson 1989). Yet, in this competition, broader considerations beyond strictly performative considerations come into play. While of growing focus and interest for the study of the bureaucracy, a measurement that is able to satisfactorily discern between the constituting dimensions of reputation, and that is validated on real‐life audiences of an actual public sector agency, has remained elusive thus far.

Important steps have been made toward developing a public sector scale. Lee and Van Ryzin (2019) developed a reliable measure for reputation by surveying citizens about three public agencies. Interestingly, the authors do not empirically find the separate dimensions predicted by Carpenter (2010), but they do find a measure with high internal consistency that does not discriminate among the different dimensions of reputation. In contrast, the current contribution empirically demonstrates the presence of separate dimensions of bureaucratic reputation by surveying multiple audiences of a regulatory agency. The use of different audiences allows us to venture beyond the work on citizen attitudes such as trust in government organizations (e.g., Grimmelikhuijsen and Meijer 2014). At the same time, our contribution shows that to identify the different dimensions, stakeholders need to be sufficiently familiar with the organization. The development of a measurement using a sample of stakeholders that directly engage with the agency allows us to empirically test and validate the multidimensionality of the bureaucratic reputation concept.

This is precisely the contribution of this article: we develop an empirical tool that is able to measure the reputation of a public agency and explicitly sets out to identify and measure its subcomponent dimensions. By discerning these different reputational dimensions, the development of such a scale makes an important contribution to the literature, as it will allow reputation scholars to study (and measure) which aspects organizations are “known for” and build their claim to authority on, as well as how the profiles of public organizations differ in this respect. We build our survey instrument on the theoretical groundwork laid out by Carpenter (2010) and deductively generate an item pool. To develop, test, and cross‐validate the scale, we carried out two surveys with 2,100 key stakeholders of the European Chemicals Agency (ECHA) in Helsinki—the European Union (EU) chemicals regulator.

The proposed instrument furthers the development of bureaucratic reputation theory, as it demonstrates the generalizability of the constituting dimensions of reputation *across* an agency's various audiences. It also establishes the added value of a public reputation measurement beyond existing measurements in the private sector. This study also has important implications for practice. The reputation literature demonstrates that future organizational interactions and perceptions of organizational competence and authority are informed by past experience (Carpenter 2001). While public organizations increasingly engage in reputation management activities, a potential caveat that comes out of our exercise is that managers of public organizations might be steering in non‐astute directions. While our study shows that, as for private actors, “performance matters,” procedural and moral aspects weigh heavily as well when it comes to public regulators. To secure a positive organizational image and the authority crucial for public regulators to operate, the performance management turn in the public sector may need to be supplemented by enhanced organizational attention to procedural and moral aspects.

## Reputation in the Public Sector

Bureaucratic reputation has become an established lens through which to study public sector organizations, with its proponents pointing to its greater explanatory power over classic theories of regulation such as public interest or capture theories for understanding regulation and the behavior of regulators (Carpenter 2010, specifically pp. 35–45 on this point). What is more, with its transactional (Carpenter and Krause 2015), as opposed to hierarchical, understanding of regulatory authority, this lens proposes an alternative theoretical account to dominant principal‐agent models of bureaucratic politics.

As noted earlier, the relevance of reputational concerns has been demonstrated in driving a variety of regulatory behaviors, including the supply of regulatory outputs (Maor and Sulitzeanu‐Kenan 2016), the duration of drug approval (Carpenter 2002) or of enforcement decisions (Maor and Sulitzeanu‐Kenan 2013), enforcement practices (Etienne 2015), regulatory risk assessment practices (Rimkutė 2018), turf management and regulatory cooperation (Maor 2010; Moynihan 2012), strategic use of communications (Gilad, Maor, and Bloom 2015; Maor, Gilad, and Bloom 2013), bureaucratic demand for public participation (Moffitt 2010, 2014), and accountability and political control (Busuioc and Lodge 2016, 2017). Moreover, the value of the reputational perspective has been demonstrated not only in the regulation context but also with respect to public sector organizations in a variety of forms and contexts, including health care (Wæraas and Sataøen 2015), higher education (Christensen and Gornitzka 2017; Christensen, Gornitzka, and Ramirez 2019), social security (Christensen and Lodge 2018), police and border management (Busuioc 2016; Christensen and Lægreid 2015), and different levels of government—from municipal organizations (Lockert et al. 2019; Wæraas 2015) to ministries and/or national‐level departments (Lee and Van Ryzin 2018; Luoma‐Aho 2007). To an overwhelming degree, empirical studies in this tradition have tended to be either qualitative in‐depth case studies that try to reconstruct reputational processes (e.g., drawing on thick description, historical data, and/or interview data) or quantitative studies that focus on measuring specific reputational aspects (such as “reputational threats” and regulatory responses) rather than organizational reputation as such: “None of the current bureaucratic reputation scholars measure reputation per se, but rather reputational threats as manifested in the media” (Maor 2016, 86).

Reputation refers to the external image of an organization. It is understood as “symbolic beliefs about an organization—its capacities, intensions, history, mission—and these images are embedded in a network of multiple audiences” (Carpenter 2010, 33). Its key elements, in other words, are perceptions of unique organizational capacity, embedded in audiences. Successful reputation building emerges as a result of organizational establishment of *durable* links with the political and social environment. In Carpenter's historical account, reputation comes about as a product of demonstrated capacity to local communities and their networks (through successful and incremental program innovation), as well as growing “mutual familiarity” and interaction with Congress, facilitated during the Progressive Era by longer bureau chief tenures and congressionally enhanced committee time investments: “the abilities and the interests of bureaus became clearer … and uncertainly over the bureaucracy declined” (Carpenter 2001, 363).

The concept of *audiences* is central to bureaucratic reputation theory and sets it apart, for instance, from studies that measure citizen attitudes toward bureaucracy. Organizations are embedded in *multiple*, durable *networks* of *audiences* that are both broader/more multiple than citizens (encompassing regulatees, professional networks, etc.) as well as simultaneously more specified—not all audiences are equally consequential in their ability to shape and define the reputation of an organization.

A defining characteristic of bureaucratic reputation, beyond its multiplicity, is its *multidimensionality*: “an agent can have more than one reputation, as in a reputation for discipline among one's co‐workers and a reputation for charity in one's residential community” (Carpenter 2010, 57).^1^ Reputation is not a unidimensional concept but one that draws on multiple bases. These bases are consequences of the goal ambiguity of public organizations and of the different nature of the objectives and challenges that public organizations face. In contrast with private corporations, where profit is the ultimate goal and reputation is objectified by a stock market value, for public organizations, various aspects of the public interest become salient to their reputation. Agencies not only need do their job right, they also need to do the right thing. And they need to do so within the boundaries of the law (e.g., the legal constraints of their mandates and/or administrative law requirements). Canonical literature on the bureaucracy identifies expertise as both the source of bureaucratic power (i.e., informational asymmetries) and its source of legitimation, but the sources of reputation go beyond expertise alone. Carpenter (2010) identifies four dimensions of organizational image, which include but are not restricted to expertise.

The *performative dimension* of an organization's reputation rests on its ability to undertake effective action and deliver on its mandate and declared policy priorities. Having a reputation for high performance means that the organization is perceived by its audiences to deliver on its promises, to provide high‐quality output/decisions, and to have demonstrated unique added value to its audiences, above and beyond other organizations in the field. An organization that performs is perceived as competent and effective by its environment.

The *technical or professional* dimension of reputation pertains to an organization's technical skill, analytical capacity, and/or methodological competency. Organizations with high technical reputations experience deference to their decisions and set the standard (e.g., methodologically, substantively) for other regulators in their field. This dimension of reputation has the closest links with the literature on bureaucratic politics, where expertise is considered the key source of legitimation and power of public agencies.

The *legal‐procedural dimension* of reputation is informed by the image of the organization abiding by procedural standards and due processes. This dimension of performance highlights the relevance of following accepted procedures in carrying out regulatory tasks ranging from risk assessment to regulatory decision‐making processes. Having a high reputation on this dimension would entail, among other things, that its audiences perceive the agency's decisions as not arbitrary, that due process is followed, that inclusion/exclusion of evidence follows standard procedures, that conflicts of interest are adequately dealt with so as to avoid capture by business or other vested interests, and so on.

The *moral dimension* of reputation refers to an agency's commitment to moral and ethical values and standards in terms of both its mandate and its actions. Standards include regulatory transparency, compassion, protection of citizens from harm, acting in the public interest, ethical behavior, integrity, flexibility to constituency needs, and so on (Carpenter 2010), as assessed by audiences. Having a high moral reputation is important for public organizations, as protecting the public interest and having a positive influence on society legitimize their existence and set these organizations apart from corporations.

A reputational lens recognizes that assessments can vary across audiences, as different audiences come with different visions of the organization (“what one audience sees is not necessarily what another audience sees”; Carpenter 2010, 34) as well as different expectations of it. Organizational assessments are not objective but rather a matter of perception, where different audiences have different standards and expectations.

While most authors studying reputation in the public sector draw on Carpenter's definition, and a coherent body of work is developing as a result, some conceptual departures from this are also emerging. For instance, some authors have emphasized the relevance of additional dimensions emerging from empirical work: Salomonsen, Verhoest, and Boye (2018) identify the relevance of a *processual* dimension of reputation, in addition to the four dimensions discussed earlier; Lee and Van Ryzin (2019) compile all of the dimensions into a single measurement with a high reported internal consistency; and Capelos et al. (2016) speak of two reputational components: a reputation *for efficacy* and *for morality*. Given the prevalence and influence of Carpenter's theoretical framework, however, in the development of our scale, we explicitly set out to identify and measure the four dimensions of reputation as identified in his work.

## Method

Our study is methodologically innovative because we surveyed stakeholders of direct relevance to the organizations we focused on—which were identified (upon our request) by the agency as relevant. Since reputation is a relational concept and implies transactional relationships between an organization and its social/political environment, examining a broad population sample may provide only a fuzzy view of what a public organization's reputation really looks like. Our study provides a close picture of the relevant constituent parts of reputation for a public organization because it surveys individuals who have a relatively closer knowledge and experience of interaction with the agency and whose views are crucial for the organizational success of the agency. Table 1 provides an overview of descriptive statistics for our respondents.

**Table 1:** Response Frequencies

		Wave 1 (*N* = 361)	Wave 2 (*N* = 347)
Age			
18–24	2	1	
25–34	43	46	
35–44	88	90	
45–54	86	93	
55–64	94	74	
65–74	25	21	
75–84	2	1	
> 85	0	1	
Not specified	3	19	
Gender			
Male	150	190	
Female	190	136	
Not specified	3	21	
Education			
Less than high school	1	3	
High school graduate	11	14	
Some higher education, no degree	16	12	
Bachelor's degree	57	58	
Master's degree	139	135	
Professional degree	29	28	
Doctorate	86	77	
Not specified	4	20	
Contact frequency with ECHA			
Never	46	57	
Less than once a year	80	71	
1–5 times per year	83	92	
6–11 times per year	31	36	
Monthly	38	34	
Weekly	55	38	
Daily	9	3	
Not specified	1	16	
Employment sector			
EU Institutions and bodies	7	3	
International organizations	6	3	
National government	31	31	
Local/regional government	3	8	
Nongovernmental organization	7	6	
Legal	2	2	
Academic	4	4	
Consultancy	69	55	
Manufacturing industry	193	179	
Distribution and logistics	11	16	
Health	5	4	
Media	1	4	
Other	19	13	
Not specified	3	19	

As we aimed to stay close to Carpenter's approach, we similarly focused on a regulatory agency.^2^ Reputational patterns are argued to be particularly pertinent for agents of social and economic regulation. Often characterized by a “diminutive staff,” while “charged with enforcing complex statutes over wide spaces of territory and technology” (Carpenter 2010, 34), the successful cultivation of organizational image becomes crucial to de facto authority and the ability to secure deference to (enforcement) decisions. In that capacity, regulatory agencies constitute a critical case for bureaucratic reputation in the public sector as a whole. Reputation is their main source of legitimacy and informs their capacity to act. Other parts of the public sector may exhibit similar patterns insofar their capacity to act depends on their reputation.

Carpenter analyzed the U.S. Food and Drug Administration. We chose a corresponding regulatory agency at the EU level, the European Chemicals Agency. ECHA is an EU‐level technical body that, together with national authorities (chemical regulators of the EU member states), plays a central role in the implementation of the EU Chemicals Regulation (REACH)—the EU‐wide regulation on chemical substances. In that context, ECHA receives and evaluates individual industry registrations of chemical substances for their compliance with regulatory standards and determines whether the risks identified can be adequately managed. In this context, ECHA possesses formal decision‐making powers—it has the power to adopt decisions that are binding on third parties—which renders it among the more powerful EU regulatory agencies, although EU agencies are not as powerful as their U.S. counterparts (Busuioc 2013).

ECHA has a broad array of stakeholders, including a variety of industry bodies, EU institutional actors, and other EU agencies; corresponding national regulatory bodies; national political actors; and citizens. Importantly, it is not an agency that is especially controversial, and it has not suffered major incidents in its lifetime. In fact, it has become a role model for non‐EU countries in implementing the REACH chemical regulations program with identifiable success (Biedenkopf 2015). This was important, we felt, as sudden incidents, crises, or particular controversies might tilt the development of the measurement instrument in one particular direction and hinder the development of an adequate and generalizable measure.

We developed the measurement instrument largely in accordance with recommendations by Hinkin (1998). He suggests a six‐step strategy consisting of item generation, pilot questionnaire administration, item reduction, confirmatory factor analysis, validity assessment, and replication. The first wave of the survey took place in September 2017, and a second survey of stakeholders took place in November–December 2017.

## Developing a Multidimensional Measurement Model

### Item Generation and Pilot Survey

We started by deductively generating an item pool based on Carpenter's (2010, 46–47) four dimensions of reputation. For each of the four dimensions, the authors formulated about 10 statements, which were intended to tap into the latent construct. In comparison with the work of previous scholars, including Ponzi, Fombrun, and Gardberg (2011) and Lee and Van Ryzin (2019), we formulated items that would ideally relate to a single dimension, rather than to an overall reputation, which were subsequently tested. Table 2 presents the initial pool of 41 items, which were presented to respondents in random order. All items had 7‐point answer categories ranging from “strongly disagree” to “strongly agree.”

**Table 2:** Initial Item Pool

Dimension	Item
Performative	The Agency has sufficient capacity resources, personnel, capital to deliver on its mandate.
	**The Agency**'**s output is of high quality.**
	**The Agency is an effective organization.**
	**The Agency does what it promises.**
	It would be difficult for another organization to do the job the Agency carries out.
	Replacing the Agency with any other organization would deteriorate the current level of service quality.
	**The Agency has a lot of added value.**
	**The Agency is innovative and entrepreneurial in solving problems.**
	**The Agency makes good decisions.**
	**The Agency is a competent regulator.**
	**The Agency communicates well with stakeholders.**
Moral	**The Agency**'**s mission is ethically defensible (their mission is the right mission).**
	**The way in which the Agency works is ethically defensible.**
	**Outputs (e.g., decisions, rules, opinions, products) of the Agency are ethically defensible.**
	**The Agency has a positive influence on society.**
	The Agency shows compassion toward people or organizations that are disadvantaged by its actions.
	**The Agency has integrity.**
	**The Agency works transparently.**
	**Confidentiality is an important value for the Agency.**
	**The Agency pays attention to public opinion.**
	The Agency is independent from political considerations.
Technical	**The Agency**'**s employees are highly skilled in their profession.**
	**The Agency**'**s employees understand the problems and issues in the field.**
	**The Agency cooperates well with experts in the field.**
	**The Agency is a learning organization.**
	The Agency has good leadership.
	Expertise in the Agency is well managed, even if an employee leaves.
	The Agency has the capacity to maintain qualified staff.
	**The Agency sets new scientific standards.**
	The Agency is at the forefront of scientific innovations.
	**Opinions by the Agency influence what we do in our own organization.**
Legal‐procedural	**Decision‐making in the Agency follows due process.**
	The Agency works fairly and does not make arbitrary decisions.
	The Agency has a good procedure for complaints.
	**The Agency uses all of the relevant evidence.**
	**The Agency follows correct procedures.**
	**The Agency**'**s experts are objective.**
	The Agency strikes a good balance between transparency and confidentiality.
	Most opinions by the Agency do not get challenged.
	**The Agency is good at responding to requests for information in an orderly and timely manner.**
	**The Agency is independent from industry.**

Note: Items in boldface were retained for further analysis.

Upon our initiative and at our request, the agency identified around 2,100 individual stakeholders of the organization. These stakeholders were divided into 13 categories based on the employer type (e.g., academia, other EU institutions, consultancies, industry, etc.). We developed an online survey and included a nonidentifying link in the invitations to preserve respondents’ anonymity.

For the first survey, the agency selected a stratified random sample of 50 percent, such that invitations would be sent out equally across the categories. A total of 1,082 emails were sent out by the agency, with 20 bounces. The invitation email explicitly stipulated that the survey was part of a scientific study in which the agency had agreed to participate. The authors signed the email and provided their institutional email addresses for further contact (see appendix S1 in the Supporting Information). We instructed the agency when to send out reminders and collated responses independent of the agency. A first reminder was sent two days after the original invitation (Crawford, Couper, and Lamias 2001), and two more reminders were sent 10 and 17 days after the original invitation. Moreover, the survey was announced twice in the agency's weekly bulletin (Porter 2004). Of the remaining 1,062 invited respondents, 486 opened the survey link, and 361 completed the survey, for a response rate of 34 percent. We removed six responses based on straight‐lined answers.

### Item Reduction and Exploratory Factor Analysis

We reassessed the item quality based on answering patterns in the distribution of responses (Clark and Watson 1995). Some items had a bimodal answer distribution with one of the peaks at the neutral answer option. These items might have been difficult for respondents to answer. Therefore, we reevaluated the item wording. Based on this reevaluation, we dropped 14 items that might have been difficult for respondents to answer, as these dealt with, for example, internal processes of the organization.^3^ The items in boldface in table 3 are those retained in further analysis.

**Table 3:** Exploratory Factor Analysis Three‐Factor Solution

Item	Question	Performative Reputation	Moral Reputation	Legal‐Procedural Reputation
P1	**ECHA**'**s output is of high quality.**	**0.609**		
P2	**ECHA is an effective organization.**	**0.668**		
	ECHA does what it promises.			
P3	**ECHA has a lot of added value.**	**0.794**		
P4	**ECHA is innovative and entrepreneurial in solving problems.**	**0.673**		
P5	**ECHA makes good decisions.**	**0.473**		
P6	**ECHA is a competent regulator.**	**0.562**		
	ECHA communicates well with stakeholders.			
M1	**ECHA**'**s mission is ethically defensible (their mission is the right mission).**		**0.600**	
M2	**The way in which ECHA works is ethically defensible.**		**0.745**	
M3	**Outputs (e.g., decisions, rules, opinions, products) of ECHA are ethically defensible.**		**0.854**	
M4	**ECHA has a positive influence on society.**		**0.424**	
M5	**ECHA has integrity.**		**0.404**	
M6	**ECHA works transparently.**		**0.429**	
	Confidentiality is an important value for ECHA.			0.420
	ECHA pays attention to public opinion.			
	ECHA's employees are highly skilled in their profession.			0.571
	ECHA's employees understand the problems and issues in the field.			
	ECHA cooperates well with experts in the field.			0.478
	ECHA is a learning organization.			
	ECHA sets new scientific standards.	0.435		
	Opinions by ECHA influence what we do in our own organization.			
L1	**Decision‐making in ECHA follows due process.**			**0.825**
L2	**ECHA uses all of the relevant evidence.**			**0.459**
L3	**ECHA follows correct procedures.**			**0.618**
L4	**ECHA**'**s experts are objective.**			**0.410**
L5	**ECHA is good at responding to requests for information in an orderly and timely manner.**			**0.476**
	ECHA is independent from industry.			
	Reliability	α: .91	α: .91	α: .88

Notes: Extraction method: Maximum likelihood; oblimin rotation. Cumulative explained variance: 45 percent. *N* = 174. Displaying loadings > .4.

Following this initial item evaluation, we randomly split the data from the first survey into two subsamples of equal size. With the first subsample, we conducted exploratory factor analysis, as recommended by Hinkin (1998). We calculated the polychoric correlations between the items, which allowed us to calculate correlations of categorical items. Based on both the Kaiser criterion and parallel factor analysis, the optimal number of factors for these data is four. However, maximum likelihood polychoric exploratory factor analysis with four factors yields one factor with only one of the factor loadings larger than 0.4. Therefore, we preferred a three‐factor solution. This decision was supported by a lower BIC for the three‐factor solution and only a marginally higher cumulative explained variance for the four‐factor solution (1 percent).

The three‐factor solution is presented in table 3. The solution shows three distinct factors, with factor 1 representing performative reputation, factor 2 representing moral reputation, and factor 3 legal‐procedural reputation. These factors partly reflect our theoretical presumptions, which supports the content validity of the measurement instrument. We did not find empirical support for the existence of a fourth factor. Items assumed to load on the fourth factor, technical reputation, loaded on the performative and legal‐procedural dimensions. To balance content validity and discriminant validity, we decided to drop the items that we developed for the professional reputation dimension. We retained items with factor loadings greater than .4. The items that were retained for further analysis are numbered and appear in boldface in table 3, along with their factor loadings.

### Confirmatory Factor Analysis

Cross‐validation, refinement, and improvement of a measurement instrument can be achieved through confirmatory factor analysis (Kline 2015). We used the second split half for this purpose (*N* = 171). First, we checked the model fit and reliability for the full model. We then refined and improved the model by dropping redundant items. We used the lavaan package in R to analyze the models (Rosseel 2012).

We used model fit indices RMSEA (root mean square error of approximation) and SRMR (standardized root mean residual) and incremental fit indices CFI (confirmatory fit index) and TLI (Tucker‐Lewis index). RMSEA values lower than .06 and an upper bound of the 90 percent confidence interval of .08 are generally considered indicative of good fit, as are SRMR indices of .05 and lower (Hooper, Coughlan, and Mullen 2008). For the incremental fit indices (CFI and TLI), values above .90 indicate moderate fit, and values above .95 indicate good model fit (Hu and Bentler 1998). The full model had an SRMR of .051, RMSEA of .082 (90 percent CI [.068, .096]), CFI of .926, and TLI of .913. These model fit indices indicate a low to moderate fit.

To refine and improve the model, we removed item L5, which had low communality with the latent factor, as indicated by *R* ^2^ < .40. The remaining items shared at least 42 percent of their variance with their designated factor. Based on the modification index and the expected parameter change (Saris, Satorra, and van der Veld 2009), we removed items P3, M4, M5, and M6. These items cross‐loaded on two dimensions, and removing them improved the model fit as well as the discriminant validity of the three‐factor model. Table 4 presents the final model and the standardized factor loadings. The final model had an acceptable to good fit (RMSEA = .072 and 90 percent CI [.050, .094]; SRMR = .043; CFI = .963; TLI = .952).^4^ The final model performed significantly better than an orthogonal model (CFI = .761; TLI = .707; Δχ^2^(3) = 250.58, *p* < .001). The reliability of the scale was excellent, with Cronbach's alpha for the three factors of .87, .84, and .84, respectively. The average variance extracted was indicative of convergent validity (performative reputation, .60; moral reputation, .67; legal‐procedural reputation, .60). To confirm discriminant validity, we compared the final model with a single‐factor model. The final model performed significantly better than the single‐factor model (CFI_1‐factor_ = .857; TLI_1‐factor_ = .825; Δχ^2^(3) = 133.06, *p* < .001).

**Table 4:** Confirmatory Factor Analysis Three‐Factor Solution

Item	Question	Performative Reputation	Moral Reputation	Legal‐Procedural Reputation
P1	ECHA's output is of high quality.	0.78		
P2	ECHA is an effective organization.	0.82		
P4	ECHA is innovative and entrepreneurial in solving problems.	0.65		
P5	ECHA makes good decisions.	0.85		
P6	ECHA is a competent regulator.	0.74		
M1	ECHA's mission is ethically defensible (their mission is the right mission).		0.69	
M2	The way in which the Agency works is ethically defensible.		0.91	
M3	Outputs (e.g., decisions, rules, opinions, products) of ECHA are ethically defensible.		0.84	
L1	Decision‐making in ECHA follows due process.			0.67
L2	ECHA uses all of the relevant evidence.			0.83
L3	ECHA follows correct procedures.			0.72
L4	ECHA's experts are objective.			0.81
	Reliability	α: .87	α: .84	α: .84
		AVE: .60	AVE: .67	AVE: .61

Notes: RMSEA = .072 and 90% CI [.050, .094]; SRMR = .043; CFI = .963; TLI = .952. *N* = 171.

### Replication

We administered the questions from table 4 to a second sample of ECHA stakeholders to validate the instrument. Cross‐validation of the model is an essential step in the development of measurement models. This prevents capitalization on chance and improves the external validity of the model. We added two items in the moral and technical dimensions to ascertain the absence of a four‐factor structure, which did not influence our conclusions. In the second wave, a survey was sent to the remaining 1,064 stakeholders of the organization, following a similar pattern of invitations and reminders as in the first wave. Of this second sample, 347 respondents completed the survey, for a response rate of 33 percent. In this validation sample, we found that the factor structure was less clear than in the first wave.

In particular, we found that correlations between factors were high. For the full model, as in table 4, we found a model fit that was acceptable (RMSEA = .066 and 90 percent CI [.056, .076]; SRMR = .036; CFI = .951; TLI = .942). Reliability is demonstrated with average value explained (AVE) above .5 and Cronbach's alphas between .68 and .86. Note that with two items, Cronbach's alpha may underestimate the reliability (Eisinga, te Grotenhuis, and Pelzer 2013). However, correlations between the latent variables were above desired levels, varying between .92 and .96. These high correlations between the constructs indicate that the discriminant validity of the measurement instrument is threatened. When discriminant validity is not sufficiently established, dropping one or more independent variables can be used as a solution (Farrell 2010). We assessed the collinear items as well as the theoretical significance of each of the items to identify the constructs. We reduced our final measurement model to seven items to measure the three dimensions: performative, moral, and legal‐procedural reputation. We used item M4 instead of items M2 and M3 to further increase discriminant validity. The items and standardized factor loadings are reported in table 5.

**Table 5:** Validation Model

Item	Question	Performative Reputation	Moral Reputation	Legal‐ Procedural Reputation
P1	ECHA's output is of high quality.	0.838		
P2	ECHA is an effective organization.	0.790		
P6	ECHA is a competent regulator.	0.821		
M1	ECHA's mission is ethically defensible (their mission is the right mission).		0.768	
M4	ECHA has a positive influence on society.		0.766	
L1	Decision‐making in ECHA follows due process.			0.695
L3	ECHA follows correct procedures.			0.731
	Reliability	α: .86	α: .74	α: .68
		AVE: .66	AVE: .59	AVE: .51

Note: Standardized factor loadings.

The reduced model performed better with discriminant validity, with the highest correlation (between performative and moral reputation) amounting to .87. This is still considered relatively high, and above the commonly used .85 threshold. However, we identified the respondents which reported to have contact (at least once a year) with the agency and retested the model (*n* = 203). The model fit is considered excellent: RMSEA = .032 and 90 percent CI [.000, .084]; SRMR = .024; CFI = .996; TLI = .991. The separate dimensions of reputation could be discriminated with respondents who are at least once a year in contact with the agency. With this subsample the maximum correlation reduced to .83, which is indicative of discriminant validity. The three‐factor model for this subsample has a better fit than the single‐factor model (CFI_1‐factor_ = .954; TLI_1‐factor_ = .931; Δχ^2^(3) = 24.20, *p* < .001), which supports the discriminant validity. This shows that those stakeholders with contact make evaluations for each of the identified dimensions, whereas stakeholders without (at least yearly) contact render a single image of the organization's reputation. In other words, contact is a necessary condition to evaluate the separate dimensions of reputation independently.

### Public Reputation and Corporate Reputation

The RepTrak Pulse scale (Ponzi, Fombrun, and Gardberg 2011) has been in use for some time to measure corporate reputation. Comparing our public reputation scale with the Reputation Pulse scores serves two goals. First, it underscores the convergent validity of the public reputation scale on the overlapping dimension (i.e., performance). Second, it simultaneously emphasizes the difference between private and public organizations in their sources of reputation. They measure corporate reputation by responses to four statements: (1) “[Company] is a company I have a good feeling about”; (2) “[Company] is a company that I trust”; (3) “[Company] is a company that I admire and respect”; (4) “[Company] has a good overall reputation” (Ponzi, Fombrun, and Gardberg 2011, 23). We tested this scale in our subsample of respondents in the second wave who indicated that they had contact with the agency at least once a year. We found the scale reliable with Cronbach's alpha = .92.

**Figure 1:**
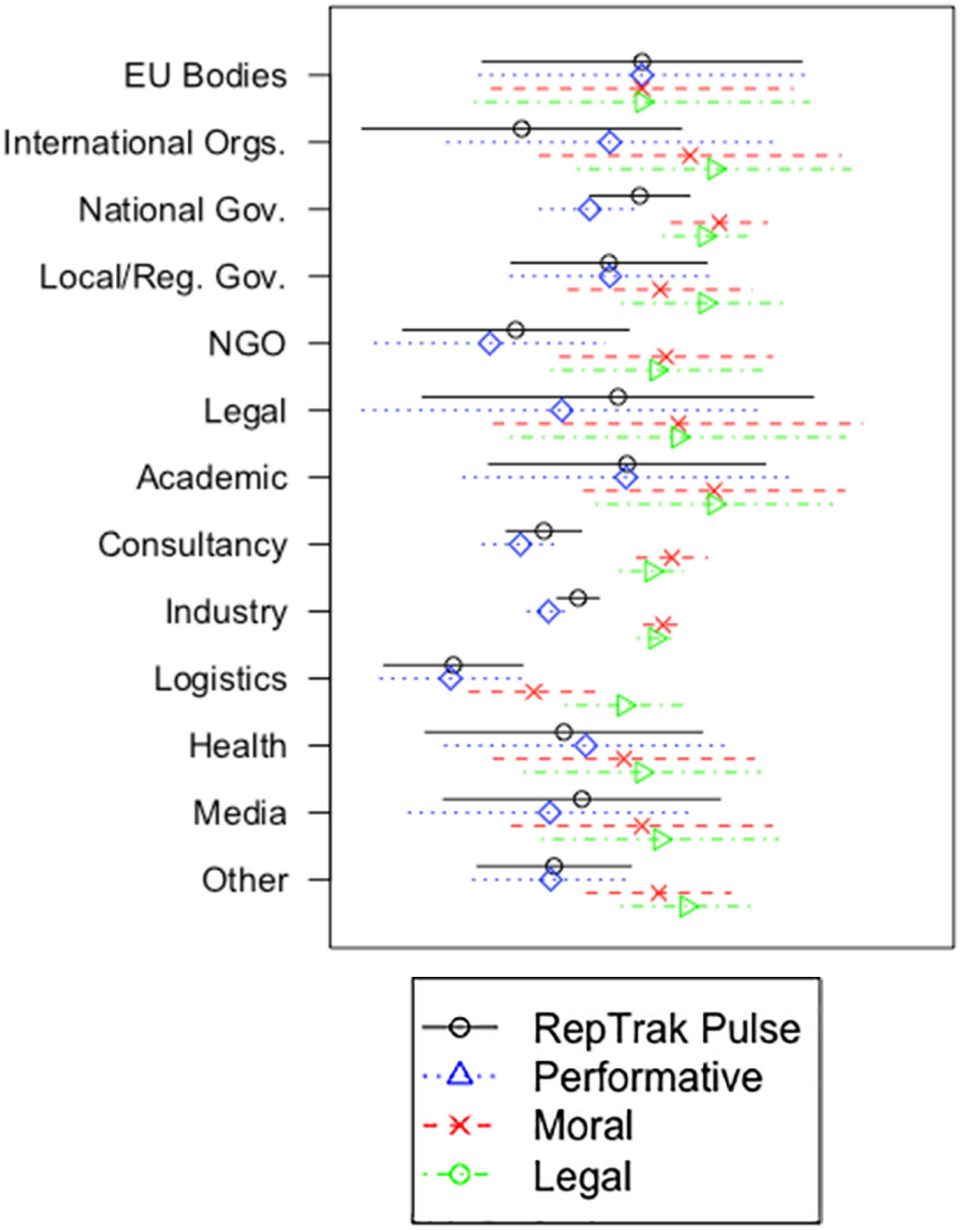
Dimensions of Reputation Compared with Reputation Pulse Scores across Stakeholder Groups Note: Estimated mean differences per dimension and 95% confidence intervals based on quasi standard errors (Firth 2003), based on data from wave 2.

To confirm convergent validity, we regressed the three factors from our public reputation scale on the Reputation Pulse scores. This model exhibited a substantive effect of bureaucratic reputation's dimensions on the Reputation Pulse scores (β_perf_ = 0.955, *p* < .001; β_moral_ = 0.834, *p* < .001; β_legal_ = 0.784, *p* < .001; all standardized weights). The regression model has a good fit: (RMSEA = .043 and 90 percent CI [.000, .069]; SRMR = .030; CFI = .989; TLI = .985). These results underscore the convergent validity of our model, but the differences in effect sizes among the separate dimensions of bureaucratic reputation also highlight the added value of a more fine‐grained measurement of organizational reputation in a public context. Reputation as measured by the Reputation Pulse scores mainly covers performative reputation. Correlations with moral and legal reputation are considerably lower. These findings are presented in figure 1, which shows the differences between the sector averages compared with EU bodies as a baseline, based on the data from the second wave. It demonstrates how averages move between sectors, and demonstrates that differences in Pulse scores mostly relate to differences in performative, but not moral or legal reputation scores.

## Analysis and Conclusion

In this article, we have developed and validated a measurement of public organizational reputation with subcomponent dimensions. We believe this represents an important step toward developing a generalized measurement instrument for gauging stakeholder perceptions of public sector organizations. A distinct merit of the approach presented here is replicability resulting from the cross‐validation of the model. Moreover, the deductive nature of our approach increases the content validity of the instrument. The study provides us with a validated measure that other studies can replicate (in different organizational, [cross‐]national, and regional contexts) and build on further.

It is, first of all, central to note that an important result of our study is that our validated barometer, in contrast with existing measurements, is able to measure *separate dimensions* of reputation. We find that a multifactor model fits better than a single‐factor model, which points to the multidimensionally of reputation in ways that Carpenter's work identifies. In the same breath, we should also note that the barometer is composed of three rather than four dimensions of reputation: we were able to identify three of the dimensions of organizational reputation predicted by Carpenter (2010). The fourth theorized dimension, technical reputation, had too much in common with another dimension to be identified separately: the technical and performative dimensions became fused in our findings and were difficult to disentangle.

This is a case of observational equivalence; two different explanations can be put forward for these findings. One explanation is that bureaucratic reputation, contrary to theoretical claims, is composed of three rather than four dimensions. A more likely explanation for our findings, in our opinion, stems from the characteristics of the public organization we focused on—a regulatory agency. Given the technical nature of regulation as a field, for a regulatory agency to “perform,” to deliver on its mandate, the regulator would generally need be a technical, expert‐based organization. In fact, the discourse on the legitimacy of the EU regulatory state and its agents (i.e., regulatory agencies such as ECHA) fuses these two dimensions: EU regulators are legitimated by their expert outputs—by their ability to produce high‐quality outputs as a result of their technical/expert‐based nature (Majone 1997, 2001). Seen in this light, our findings are not in contradiction to, but rather in alignment with, the theoretical literature. As noted by Carpenter (2010, 47), reputational dimensions are interrelated: “the different facets of organizational reputation overlap.” It becomes difficult for audiences to separate these two dimensions or even to think about them as separate elements when assessing ECHA, given the technical expertise required to “perform” in this case. In this context, it makes sense that identifying distinctive patterns associated with technical reputation—so as to allow us to disaggregate it as a separate dimension—proved challenging. The collapse of these two dimensions into a single performative dimension is likely to occur in organizations that have highly professionalized technical/expert tasks. Many public regulatory and executive agencies, nowadays, match this description, which improves the generalizability of our findings beyond the current case and other chemicals regulators worldwide.

Importantly, we further found, in line with theoretical expectations, that stakeholders that are closer to the organization are better able to discern the different dimensions of reputation. We believe this testifies to the robustness and the quality of our measurement and the value of drawing on actual stakeholder perceptions in developing a reputation measurement. The separate reputational dimensions are better identifiable by stakeholders under conditions of proximity: regulatory image comes into sharper focus for stakeholders who have more interactions with the organization.

To attempt to disentangle the performative and technical dimensions, future studies should attempt to replicate our survey questions with a *different type of public organization*, such as a routine bureaucratic organization. More specifically, cases would need to be purposely selected to encompass organizations that score differently on these two dimensions—that is, organizations that are regarded as well performing but are not high on the expertise/technical dimension (e.g., routine organizations such as tax offices; garbage collection; welfare distribution agency) or vice versa. The technical items from table 2 can be applied to identify potential differences between the performative and technical dimensions of reputation.

More broadly, beyond the overlap we encountered on two dimensions—technical/performative—the issue of dimension overlap is important to note more generally. In our efforts to devise this measurement tool, disentangling the separate dimensions was no easy feat: several of our pilot questions loaded highly on more than one factor (and were subsequently omitted as a result). Our aim was to identify (the most) salient variables as a proxy for our factors. To be able to measure *separate*, *conceptually distinct* dimensions, we necessarily eliminated questions that are potentially relevant measures for a specific dimension but simultaneously also measure other dimensions.^5^ In other words, to disentangle our dimensions, we necessarily focused on a narrow set of core measurements. We tried to measure the dimensions at the extremes of their distinctiveness, yet it seems we did not lose content validity: the performative aspect of reputation that we measure has a high correlation with the Reputation Pulse scores used to measure corporate reputation. This implies that we were able to tap into the established construct of performative reputation.

While overlap, “ambiguity and the possibility of multiple interpretations of symbols and actions” is inherent to organizational image, the advantage of a dimension‐specific rather than a cumulative measurement of reputation lies precisely in the ability to capture the *multidimensional* nature of reputation—a key distinguishing feature of the concept, also from related concepts such as legitimacy. We think this is an added value of our scale over and above measurements that do not distinguish separate factors. More specifically, our ability to measure reputation and its building blocks (subdimensions) is important for several reasons. First, it is crucial to studying organizational reputation‐building processes and legitimation processes by allowing us to “pull apart” the different dimensions that organizations come to be perceived as “standing for” and how these perceptions may differ across audiences/stakeholder groups (e.g., professional and consumer groups or institutional partners). In other words, it will allow us to study on which specific dimensions organizational claims to legitimation are based on, how the profiles of different organizations vary in this respect, and with what implications for their legitimacy. Assessing organizational image in this more differentiated and composite manner may tell us important things about how and why some agencies attain a strong reputation and authority beyond formal fiat, whereas others remain controversial.

Second, the further refinement of the measure can potentially allow us to make important headway not only in the field of organizational reputation but also to test more systematically fundamental assumptions of bureaucratic politics literature more broadly—for instance, the link between organizational reputation and autonomy. What is more, beyond its contribution to the academic literature, the measurement can be of value for practitioners, and it has value over corporate measures of reputation when it comes to their applicability to the public sector. For public organizations, our study shows, performance is not the sole standard of reputation—moral and procedural aspects find equal relevance. In a practical sense, then, public organizations cannot “afford” to only focus on performative aspects.

It is important to recognize, however, that as with any research, our study also has clear limitations. While the barometer allows us to measure, in a differentiated fashion, the perceptions of different stakeholder groups and to more specifically map out the parameters of organizational image across stakeholder groups, it does not allow us to ascertain whose visions become dominant under conflicting assessments (as a “a contest played out among diverse audiences”; Carpenter 2010, 729). Moreover, while the measurement is validated on two large‐*N* pilot studies with actual stakeholders, it is limited to one public body. However, regulatory agencies constitute a critical case for bureaucratic reputation, and ECHA provides an exemplary case of these organizations. Additional research applying the item list to other types of organizations, in different administrative (national and subnational) contexts, would be beneficial.

We believe our study makes important headway in the development of a measurement of reputation for the public sector. It simultaneously also raises interesting—and thus far unanticipated—questions that should be taken up by further studies. Particularly relevant, would be the application of our measures to other public sector organizations than regulators to observe the extent to which similar findings arise and in particular, with respect to organizations where the technical dimension would be expected to be more readily distinguishable from performative aspects.

## Funding

Sjors Overman would like to acknowledge funding received for this project from the Netherlands Organisation for Scientific Research (NWO), as part of the NWO‐vidi project “Calibrating Public Accountability” (452‐14‐008); Madalina Busuioc would like to acknowledge funding received for this project from the European Research Council (ERC) under the European Union's Horizon 2020 research and innovation program (grant agreement 716439); Matthew Wood would like to acknowledge funding received for this project from the Economic and Social Research Council (ESRC) (grant number ES/L010925/1).

## Supporting information

Appendix S1: Invitation email

## Notes

1 1.This is not unlike understandings of corporate reputation, where it has been acknowledged that firms have multiple reputations and authors have argued for a move away from overall assessments of corporate reputation to attribute‐specific ones to better account for this multidimensionality (Jensen, Kim, and Kim 2012, 143): “When reputation is viewed as attribute‐specific, an actor may simultaneously be ascribed a positive reputation with respect to a specific attribute and a negative reputation with respect to another attribute by the *same* audience. … Reputation is, accordingly, always a reputation *for* something.”

2 2.ECHA has decision‐making tasks. We should clarify that in this context, in our references to “regulatory agencies” throughout the article, we embrace a broad definition of the concept to encompass not only bodies with decision‐making, rulemaking, or enforcement tasks but also strictly information agencies, motivated by seminal EU regulation literature—for example, Majone's (1997) argument that “regulation by information” is as important a mode of regulation as direct regulation in the EU regulatory state (Majone 1997): “A good deal of empirical evidence suggests that regulation by information is often more effective than direct regulation” (Majone 1997).

3 3.We reevaluated the questions after the first survey waves and dropped 14 items, because respondents might have had difficulty responding the items. Examples of dropped items include “Replacing the Agency with any other organization would deteriorate the current level of service quality” and “ECHA has the capacity to maintain qualified staff.” The response patterns for both questions showed a bimodal distribution with local maximums on both the middle answer category (18.1 and 28.9 percent, respectively) and on the agree category (25.9 and 23.4 percent). Upon reevaluation of the questions, we feared that the hypothetical nature of first example might explain the high number of neutral answers. In the second example, respondents might not know their answer and pick the neutral option, even though no response was required to finish the survey.

4 4.RMSEA is above the recommended threshold; rather than resulting from model misspecification, this might be attributable to the relatively small samples size (*N* = 171) (Chen et al. 2008). The model fit improves when we model additional covariance between L1 and L5 (RMSEA = .055 and 90 percent CI [.028, .079]; SRMR = .043; CFI = .978; TLI = .972).

5 5.Some items loaded highly on multiple dimensions; for example, “ECHA works transparently” loaded on both moral and legal‐procedural reputation. The item “ECHA is good at responding to requests for information in an orderly and timely manner” had high loadings on both performative and legal‐procedural reputation. While these items reflect a generalized reputation, they obstruct the clear measurement of the distinctive dimensions, thereby threatening discriminant validity.
