# Supplementary material for: A Multidimensional Reputation Barometer for Public Agencies: A Validated Instrument
Source: Public Adm Rev. 2020 Feb 18;80(3):415–25. doi: 10.1111/puar.13158 (PMC7319416; doi:10.1111/puar.13158)
Supplement: Supplementary file 1 — Appendix S1: Invitation email [file PUAR-80-415-s001.docx]

Appendix S1: Invitation email

Email not displaying correctly? View it in your browser.

ECHA logo

This survey is part of an international scientific study into the measurement of reputation of public sector organisations among their stakeholders. ECHA has agreed to be part of this study.

As a stakeholder of ECHA, you have specific expertise that is invaluable to this study.

To access the survey: [link].

Your answers will be scientifically analysed to determine which questions provide the best and most reliable insights into the relationship between ECHA and its stakeholders. Please note that some questions are similar, however, we ask you to respond to all of them, as this is important for the scientific analysis.

This survey should take approximately **10** **minutes** of your time.

Your responses will remain confidential. Only anonymous and aggregated data will be used in the study and neither the researchers nor ECHA will be able to link answers to individual persons.

The study is being developed by the universities [masked],

Thank you for taking part in our survey. Your efforts are highly valuable to us.

The team responsible for this survey consists of:

[masked name]—[masked email address]

[masked name]—[masked email address]

[masked name]—[masked email address]

Unsubscribe

Contact ECHA
